# Development of Cathepsin L-like Real-Time PCR Assays for the Detection of African Animal Trypanosomosis (AAT) in South Africa

**DOI:** 10.3390/pathogens11020136

**Published:** 2022-01-22

**Authors:** Samantha Mnkandla, Luis Neves, Ilse Vorster, Raksha Vasantrai Bhoora

**Affiliations:** 1 Department of Veterinary Tropical Diseases, University of Pretoria, Private Bag X04, Onderstepoort, Pretoria 0110, South Africa; luis.neves@up.ac.za (L.N.); ilse.vorster@up.ac.za (I.V.); raksha.vasantraibhoora@up.ac.za (R.V.B.); 2 Centro de Biotecnologia, Universidade Eduardo Mondlane, Maputo 010100, Mozambique

**Keywords:** African animal trypanosomosis, cathepsin L-like, quantitative real-time PCR, cattle samples, goat samples

## Abstract

African animal trypanosomosis (AAT), is an infectious parasitic disease of wildlife and livestock caused by multiple species and strains of *Trypanosoma*. In South Africa, it is restricted to northern KwaZulu-Natal (NKZN) and caused by *Trypanosoma congolense* and *Trypanosoma vivax*. A cross-sectional study was done to determine AAT prevalence in 384 goat samples and identify trypanosome species circulating in 60 cattle at dip tanks that are on the interface with the Hluhluwe-uMfolozi game reserve in NKZN. Both cattle and goat samples were analyzed using the buffy coat technique (BCT) and a polymerase chain reaction (PCR) assay targeting the internal transcribed spacer 1 (ITS) region. Cattle samples were further analyzed using an ITS quantitative real-time PCR (qPCR) assays designed for the detection of *T. congolense*, *T. vivax,* and *T. brucei*. None of the goat samples tested positive for *Trypanosoma* infections. The ITS qPCR assay detected *Trypanosoma* DNA in 30% of the cattle samples, while only 8.3% were positive with the ITS PCR and 11.7% were positive using BCT. Quantitative real-time PCR assays were designed to amplify a 98 bp, 137 bp, and 116 bp fragment of the cathepsin L-like (CATL) gene from *T. brucei*, *T. theileri,* and *T. congolense*, respectively. Each assay was shown to be efficient (>94%) and specific (10^9^ to 10^2^/10^1^ copies/reaction) in the detection of *Trypanosoma* species. The CATL qPCR assays detected *T. congolense* and *T. theileri* infections in 33.3% of the cattle samples. The CATL qPCR assays also detected *T. congolense* infections in goats (23.1%) that were neither detected by BCT nor the ITS PCR. The CATL qPCR assays provide an additional, sensitive, and specific tool for *Trypanosoma* diagnostics. The presence of trypanosomes in goats suggests they might be potential reservoirs of infections to other livestock.

## 1. Introduction

African animal trypanosomosis (AAT) is a debilitating disease of livestock caused by extracellular flagellate protozoan parasites belonging to the family Trypanosomatidae [1]. Three species of *Trypanosoma* endemic to Africa are *Trypanosoma vivax*, *Trypanosoma congolense* and *Trypanosoma brucei* [1]. *Trypanosoma vivax* and *T. congolense* mainly infect cattle, while *T. brucei senso latum* is represented by three sub-species; *Trypanosoma brucei brucei*, which only infects animals, *Trypanosoma brucei rhodesiense*, which infects wildlife, livestock, and humans, and *Trypanosoma brucei gambiense*, which primarily infects humans, but can infect other mammals [2]. *Trypanosoma congolense*, *T. vivax,* and *T. brucei* are transmitted cyclically by tsetse flies (*Glossina* spp.), and *T. vivax* is also transmitted mechanically by biting flies (Stomoxys and Tabanids) [3,4].

In South Africa, AAT is restricted to northern KwaZulu-Natal (NKZN) covering an area of 18,000 km^2^ that makes up part of the tsetse belt [5]. The area stretches from the north of Umfolozi River to the southern Mozambique border [6] and has over 300,000 head of cattle [7,8]. The detection of trypanosomes is determined by either observing the clinical signs of animals, conducting microscopic analysis, performing serological diagnosis, or using molecular tests such as the Loop-mediated isothermal amplification (LAMP) assay, Low-flow assay, and polymerase chain reaction (PCR) assays [9,10]. Molecular diagnostic techniques such as PCR assays have better sensitivity and specificity compared to parasitological techniques [11,12,13]. However, due to their high sensitivity, quantitative real-time PCR assays allow for better detection of trypanosomes compared to parasitological techniques and conventional PCR assays [14,15,16].

Currently, diagnosis of AAT in South Africa is done through parasitological techniques and conventional PCR assays targeting either the 18S region or the internal transcribed spacer (ITS) region [17,18]. However, these diagnostic tools are time-consuming, laborious, and lack sensitivity.

The cathepsin L (CATL) genes encode for CATL-like cysteine protease in trypanosomes, which play a role in metabolism, infectivity, cell-differentiation, and pathogenicity of trypanosomes [19]. Polymorphism in the *Trypanosoma* CATL gene sequences makes them ideal targets in diagnostics, genotyping, and inferring phylogenetic relationships [19,20,21,22,23]. Conventional PCR assays targeting the CATL gene have been developed for the detection of *T. vivax* [19], *Trypanosoma rangeli* [24], and *Trypanosoma theileri* [25]. Advancing these molecular diagnostic PCR assays to real-time PCR assays would improve the detection of trypanosomes due to the high sensitivity and specificity of qPCR assays. Therefore, in this study, we describe the development of TaqMan MGB™ qPCR assays targeting the CATL-like gene for the specific and sensitive detection of *T. congolense*, *T. brucei,* and *T. theileri*.

## 2. Results

### 2.1. Parasitological Detection of Trypanosomes

Using the buffy coat technique (BCT), 7 (11.7%) of the 60 cattle were positive for trypanosomes and only *T. congolense* was detected. The highest number of infections were recorded at Mvutshini and Ekuphindisweni, where 3 (15%) *Trypanosoma-*positive animals were detected at both dip tanks. Although only one (5%) *Trypanosoma*-positive animal could be detected at Ocilwane (Table 1), there was no statistically significant difference between the infection rate of *Trypanosoma* at all three dip tanks. There was also no statistically significant difference between the average packed cell volume (PCV) of the *Trypanosoma* infected cattle as detected by BCT (27.1 ± 7.10%) and the average PCV of the uninfected cattle (30.5 ± 4.10%) (t(58) = 1.837, *p* = 0.071). Four (57.1%) of the BCT-positive cattle were anemic and 9.4% (5 of 53) of the uninfected cattle were anemic.

None of the goat samples tested positive for trypanosomes by BCT. However, the one-way ANOVA showed a significant difference of herd average PCV (HA-PCV) (F(2, 381) = 4.057; *p* < 0.05) between the three dip tanks. Goats screened at Mvutshini (n = 128) had a statistically significant higher HA-PCV (28.9 ± 5.80%, *p* = 0.025) than goats screened at Ekuphindisweni (n = 128). There was no statistically significant difference of HA-PCV between Ocilwane (28.7 ± 4.90%) and Mvutshini (*p* = 0.935), as well as no statistically significant difference between Ekuphindisweni HA-PCV (27.3 ± 3.70%) and Ocilwane HA-PCV (*p* = 0.061). Eighty-nine (23.2%) of the 384 goats sampled were anemic. There were 37 anemic goats (herd average anemia or HAA = 28.9%) at Mvutshini, 31 (HAA = 24.2%) at Ekuphindisweni and 21 (HAA = 16.4%) at Ocilwane. The independent *t*-test showed that the average PCV was significantly lower in the anemic group (22.16 ± 2.50%) than in the non-anemic group (30.17 ± 3.80%) (t(382) = −18.867, *p* < 0.0005).

### 2.2. Molecular Identification of Trypanosomes Using ITS PCR and ITS qPCR

**Figure 1:**
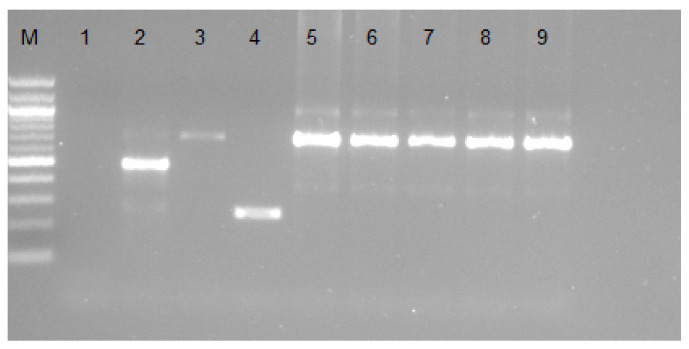
Agarose gel showing bands obtained from ITS PCR amplification using primers ITS1 CF and ITS1 BR. Lane M represents a 100 bp marker. Lane 1: negative control, lane 2: *T. brucei* positive control, lane 3: *T. congolense* positive control, lane 4: *T. vivax* positive control, and lanes 5–9 *T. congolense* positive cattle field samples.

The ITS PCR assay was used to detect *Trypanosoma* parasites in cattle and goat samples. While none of the goat samples tested positive for *Trypanosoma* parasites, 5 (8.3%) of the cattle samples (samples: 17, 32, 39, 40, and 47) produced amplicons between 620 bp–710 bp, which indicated *T. congolense* infections (Figure 1). The ITS PCR assay did not detect any *T. brucei* or *T. vivax* infections in the cattle.

The cut-off quantification cycle (Ct) value of this assay was 38 [14] and any deoxyribonucleic acid (DNA) samples with a Ct value of >38 were regarded as negative. Eighteen of the 60 cattle (30%) were positive for trypanosome parasites. Only *T. congolense* and *T. brucei* were detected by ITS qPCR and no mixed infections were observed in the cattle. Six of the 60 cattle (10%) were positive for *T. congolense* and 20% (12/60) of the cattle were positive for *T. brucei*.

### 2.3. In Silico Analysis of the Designed CATL qPCR Primers and Probes

The specificity of the cathepsin L (CATL) qPCR primers and probes designed for each *Trypanosoma* species, were each tested in silico using BLAST analysis. While the *T. congolense* and *T. theileri* probe sequences showed 100% sequence identity to published *T. congolense* and *T. theileri* sequences on GenBank, the *T. brucei* CATL probe sequence showed 100% sequence identity to *Trypanosoma evansi* and *Trypanosoma equiperdum* CATL sequences. Alignments of the designed CATL primer and probe sequences showed high sequence conservation to published *Trypanosoma* CATL sequences available from GenBank.

### 2.4. Sensitivity and Efficiency of the CATL qPCR Assays

**Figure 2:**
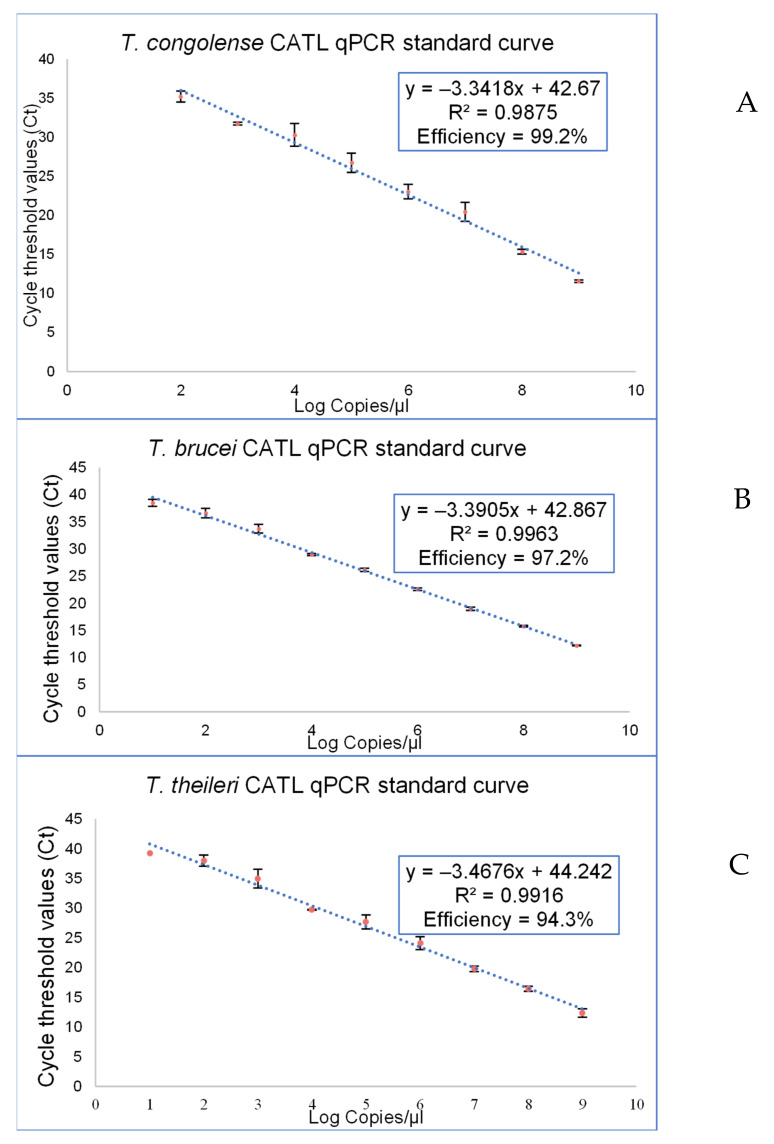
Linear regression for the quantification of (**A**) *T. congolense* (**B**) *T. brucei* (**C**) *T. theileri* parasite DNA. Cycle threshold (Ct) values were plotted against log copies/µL of the initial 10-fold dilution series of the parasite plasmid construct.

Regression analysis of each of the developed CATL qPCR assays indicated a linear correlation of log copy numbers against Ct values. The amplification efficiency in detecting parasite DNA was 99.2%, 97.2%, and 94.3%, with a correlation coefficient (R^2^) value of 0.988, 0.996, and 0.991 for *T. congolense*, *T. brucei,* and *T. theileri*, respectively (Figure 2). The *T. congolense* CATL qPCR assay could detect template DNA in the range of 10^9^ copies/reaction to 10^2^ copies/reaction, which corresponded to Ct values from 11.3 to 35.9. The amplification range for *T. brucei* extended from 10^9^ copies/reaction to 10^1^ copies/reaction with average Ct values ranging from 12.8 to 38.4. The detection limit of the *T. theileri* CATL qPCR assay was 10^2^ copies/reaction which corresponded to a Ct value of 37.9.

### 2.5. Analytical Specificity of the CATL qPCR Assays

All three CATL qPCR assays were shown to be specific in the amplification of parasite DNA from either *T. congolense*, *T. brucei,* or *T. theileri*. No amplification signals were observed from DNA isolated from other protozoal parasites known to infect cattle such as *Babesia bovis*, *Babesia bigemina,* and *Theileria parva*, other protozoal parasites known to infect cattle (Table 2). Cross-reactions between the assays were also not observed when each assay was tested against *Trypanosoma* DNA samples that included, *T. vivax*, *T. congolense*. *T. theileri* and *T. brucei* (Table 2).

### 2.6. Evaluation of Cattle and Goat Samples Using the CATL qPCR Assays

The developed CATL qPCR assays were tested for their ability to detect parasite DNA in field samples collected from both cattle and goats. Eight of the cattle samples tested positive for *T. congolense* with Ct values between 16.6 and 28.3. Thirteen field samples tested positive for *T. theileri* with Ct values between 30.7 and 37.7. Two cattle samples had mixed infections with *T. theileri* and *T. congolense*. *Trypanosoma brucei* infections were not detected in the cattle samples. None of the goat samples tested positive for either *T. brucei* or *T. theileri*, but *T. congolense* parasite DNA could be detected in 9 (23.1%) samples using the *T. congolense* CATL qPCR assay. The Ct values for the *T. congolense* positive samples ranged between 30.4 and 35.

### 2.7. Comparing Diagnostics Techniques for the Detection of Trypanosomes

Detection of trypanosomes in the 39 goats by the CATL qPCR assays was higher (9/39) than detected using either BCT or the ITS PCR assay. The CATL qPCR assays detected mixed infections in cattle, that were not detected by the other diagnostic techniques. Although CATL qPCR assays detected more infections in cattle than ITS qPCR assay (Table 3), Fisher’s exact test showed the difference was not statistically significant (*p* = 1.00). There was also no statistically significant difference between cattle infection rates detected by BCT or by ITS PCR assay (*p* = 0.762). The qPCR assays detected significantly more cattle *Trypanosoma* infections than BCT and conventional PCR. Significantly higher cattle infection rates were detected by the CATL qPCR assays than by the ITS PCR assay (*p* = 0.002) and BCT (*p* = 0.014). Similarly, significantly higher cattle infection rates were detected by the ITS qPCR assay than by the ITS PCR (*p* = 0.005) and BCT (*p* = 0.023). Twelve cattle samples were detected as *T. brucei* positive by the ITS qPCR. However, some of this *T. brucei* parasite DNA (66.7%), were subsequently identified as *T. theileri* by the *T. theileri* CATL qPCR assay. None of the *T. brucei* ITS qPCR positive samples were detected using the *T. brucei* CATL qPCR assay. The *T. congolense* CATL qPCR assay detected more *T. congolense* parasite DNA than BCT, ITS PCR assay, and the ITS qPCR assay (Table 3).

## 3. Discussion

African animal trypanosomosis (AAT) is an incapacitating disease that impacts the health of cattle, and the livelihood of small-scale farmers in NKZN. Despite no re-emerging outbreak since 1990 in NKZN [7], AAT remains an important disease that needs continuous monitoring to help improve control strategies and reduce economic damages [26].

This study aimed at determining the occurrence of trypanosome infections in cattle and identifying the different *Trypanosoma* species circulating in cattle within the NKZN region. The study also aimed at determining the occurrence of *Trypanosoma* infections in goats, and therefore the possible role that goats might have in maintaining and spreading infections. The buffy coat technique (BCT) was used as the parasitological diagnostic tool in this study due to its improved sensitivity and simplicity in detecting trypanosomes compared to other parasitological techniques [27]. The herd average packed cell volume (HA-PCV) and herd average anemia (HAA) were used as AAT indicators, as well as, to indicate the health status and productivity of animals [28]. Animals with PCV of less than 24% were characterized as anemic. Buffy coat smears of samples collected from cattle in NKZN were used to determine the presence of trypanosomes at communal dip tanks [8,29]. Gillingwater et al. [29] reported infection rates of 0.9%, 9.4%, and 0% at the communal dip tanks Ekuphindisweni, Mvutshini, and Ocilwane, respectively. Subsequent studies done by Ntantiso et al. [8] showed increased infection rates of 8.9%, 12.3%, and 2.9%, respectively within these dip tank areas due to the lack of control strategies. The current study shows that the infection rate is even higher using BCT at Ekuphindisweni (15%), Mvutshini (15%), and Ocilwane (5%) than reported by both Gillingwater et al. [29] and Ntantiso et al. [8]. It was further observed that the HA-PCV of both infected and non-infected cattle were above the 24% cut-off, thus indicating that the cattle herds were in good health. In contrast, the HAA of infected cattle (57.1%) was significantly higher than the HAA (9.4%) observed for non-infected cattle, indicating that anemia in infected cattle was a result of *Trypanosoma* infections. Some of the infected cattle had high PCV values, and were consequently non-anemic, suggesting low parasitemia in these positive cattle. Anemia in the uninfected cattle suggests the presence of confounding factors such as poor nutrition or helminth infections [28]. The observed HAA and HA-PCV results indicate that AAT detected in this study has an impact on the productivity of infected cattle.

In a study conducted in India to determine the prevalence of *Trypanosoma evansi* in cattle and buffaloes, both conventional parasitological and molecular methods of detection were compared [16]. Their study showed that qPCR assay targeting the ITS1 rRNA gene was far more sensitive in detecting parasite DNA. Another study compared detection of *T. evansi* in experimentally infected mice using real-time PCR assay and the hematocrit centrifugation assay [15]. The study showed the qPCR assay had better sensitivity and was faster in detecting trypanosomes [15]. In the current study, the ITS qPCR assay detected *Trypanosoma* parasite DNA in 30% of the sixty cattle sampled. The ITS qPCR assay detected 61.1% and 72.2% of *Trypanosoma* infections that were not detected by BCT and ITS PCR, respectively. These results are comparable to the findings by Silbermayr et al. [14], who also observed that the ITS qPCR assay had better sensitivity and specificity than the ITS PCR.

**Figure A1:**
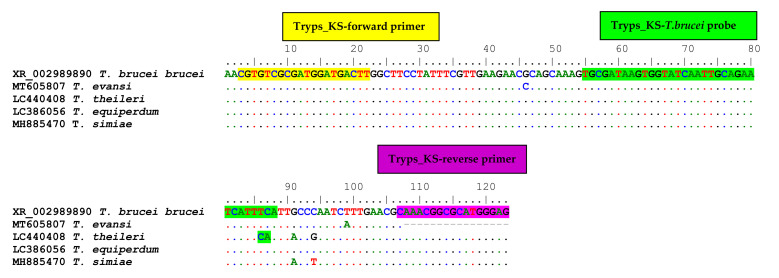
Nucleotide alignment of a section of the ITS gene of published *Trypanosoma* sequences (*T. brucei brucei*, XR_002989890; *T. evansi*, MT605807; *T. theileri*, LC440408; *T. simiae*, MH885470. The sequences of the Tryps_KS-for primer sequence are highlighted in yellow, the Tryp-KS-reverse primer sequence is highlighted in pink, and the Tryps-KS-*T. brucei* probe sequence is highlighted in green. Nucleotide differences between *Trypanosoma* species in the probe region are highlighted in the same color used to indicate the probe sequence.

Trypanosomes detected by the ITS qPCR assay, in the current study, were *T. congolense* and *T. brucei*. The identification of *T. brucei* as the most abundant trypanosome (20%) is very strange because it is a rare parasite in South Africa. *Trypanosoma brucei* had not been recorded in NKZN for several years [17,30,31,32]. However, Taioe [18], subsequently detected *T. brucei* in tsetse flies (*Glossina brevipalpis*) in the same region. The study used a nested PCR targeting the glycosomal glyceraldehyde-3-phosphate dehydrogenase (gGAPDH) gene which identified 4 sequences with high homology to *T. brucei*. The author suggested that the detection of *T. brucei* indicates that there is a possibility that these parasites have remained undetected due to the lack of sensitivity of available diagnostic assays [18]. However, molecular analysis in prior studies only detected *T. congolense* in tsetse flies [17,29]. It has been reported that the non-pathogenic parasite, *T. theileri* occurs globally and can sometimes confuse diagnostic techniques [33,34]. For these reasons, we speculated that the *T. brucei* probe region in the ITS qPCR assay was cross-reacting with *T. theileri* DNA, resulting in the observed false-positive results. Blast analysis of the ITS qPCR *T. brucei* probe showed 100% sequence identity to published sequences from *T. evansi*, *T. equiperdum*, *T. simiae*, *T. brucei,* and *T. theileri* (Appendix A: Figure A1), confirming that the ITS qPCR assay developed for the detection of *T. brucei* does cross-react with other *Trypanosoma* species and therefore lacks specificity. Consequently, the CATL TaqMan MGB™ qPCR assays were designed to differentiate between *T. brucei*, *T. theileri,* and *T. congolense* infections and to confirm whether the *T. brucei* positive samples detected were indeed false positives.

*Trypanosoma congolense* parasite DNA could be detected in 10% of cattle sampled across all three dip tanks. There was a strong correlation between the ITS PCR and ITS qPCR in the detection of *T. congolense*, as the qPCR assay detected all the *T. congolense* infections that were detected by the ITS PCR, with an additional *T. congolense* positive sample detected at Ekuphindisweni. *Trypanosoma vivax*, together with *T. congolense*, was responsible for the 1990 AAT outbreak in NKZN which killed over 10 000 cattle [7]. Subsequent prevalence studies reported these two trypanosomes as the major AAT causative parasites, with *T. vivax* accounting for very few infections [17,30,31]. Studies that followed were, therefore, not inclusive of *T. vivax* and only reported the prevalence of *T. congolense* [8,29,35]. The current study did not detect any *T. vivax* infection by both the conventional ITS PCR and the ITS qPCR assays. A study that was conducted in NKZN to determine the *Trypanosoma* prevalence in the area also did not detect *T. vivax* infections in cattle using a PCR targeting the ITS regions [18]. However, a much larger sampling size, that is inclusive of game animals, bovines, and small ruminants are needed to understand this parasite’s potentially new distribution pattern or the possibility of its absence in the region.

To improve the detection and differentiation of *Trypanosoma* species infecting livestock (AAT), we looked at developing a molecular assay targeting the CATL gene which has been reported as a suitable marker for species discrimination [19,25]. The CATL-like gene has been previously targeted, in PCR assays, to detect *T. vivax* [15,19], *T. rangeli* [24], and *T. theileri* [25]. In this study, the developed qPCR assays based on CATL for each species showed excellent linearity and high specificity, and that there was no cross-reactivity between *Trypanosoma* species tested. The detection limits ranged from 10^9^ to 10^2^/10^1^ copies/µL and the efficiencies ranged from 94–100%. In silico BLAST analysis of each of species-specific probe sequences indicated 100% sequence homology to *Trypanosoma*. The *T. congolense* probe sequence showed 100% sequence identity to published *T. congolense* sequences on GenBank. The *T. brucei* CATL probe sequence, however, showed 100% sequence identity to *T. evansi* and *T. equiperdum* CATL sequences. This is not surprising as *T. evansi*, *T. brucei* and *T. equiperdum* belong to the Trypanozoon subgenus. The *T. brucei* CATL qPCR assay could potentially be used in the detection of parasites belonging to the Trypanozoon subgenus. The *T. theileri* CATL probe sequence also showed 100% sequence identity to *Trypanosoma* (Megatrypanum) *trinaperronei*, a parasite characterized in white-tailed deer, which was shown to be phylogenetically related to *T. theileri* [36]. *Trypanosoma* research in South Africa has not focused much on *T. theileri* as it is a non-pathogenic parasite. However, *T. theileri* poses a risk when the health status of the herd is compromised by other confounding factors such as malnutrition and co-infections with other parasites [37,38]. Accurate diagnosis of trypanosomes is crucial for epidemiological studies.

**Figure A2:**
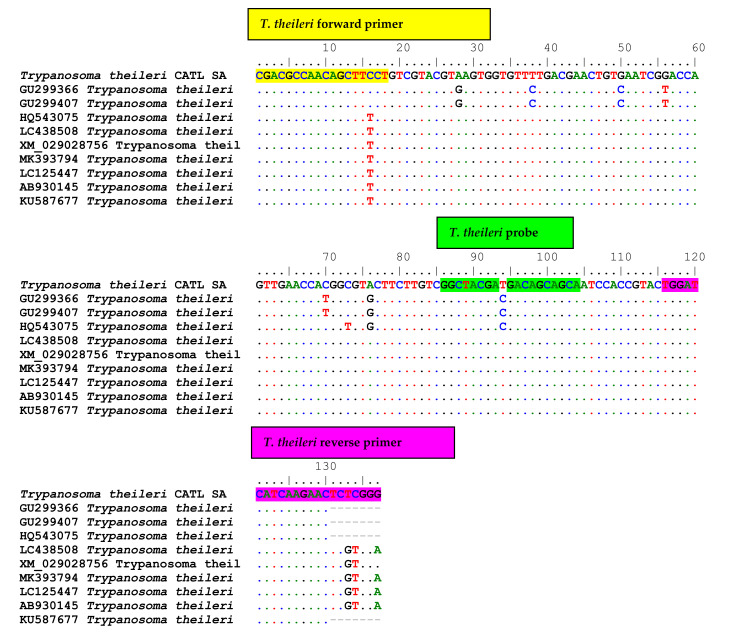
Nucleotide alignment of a section of the *T. theileri* CATL-like gene sequence against published *T. theileri* CATL-like sequences available on GenBank. The sequence of the species-specific forward primer is highlighted in yellow, while the genus-specific reverse primer is highlighted in pink and the species-specific *T. brucei* probe sequence is indicated in green.

**Figure A3:**
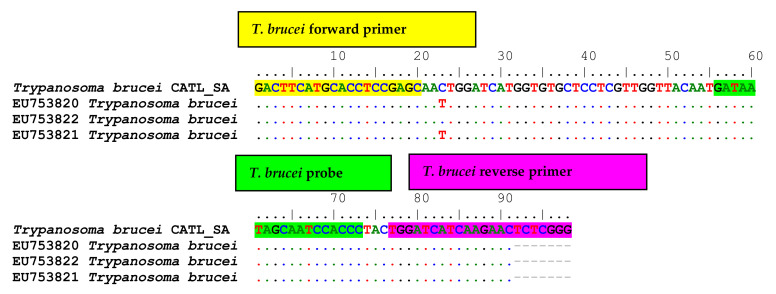
Nucleotide alignment of a section of the *T. brucei* CATL-like gene sequence against published *T. brucei* CATL-like sequences available on GenBank. The sequence of the species-specific forward primer is highlighted in yellow, while the genus-specific reverse primer is highlighted in pink and the species-specific *T. brucei* probe sequence is indicated in green.

**Figure A4:**
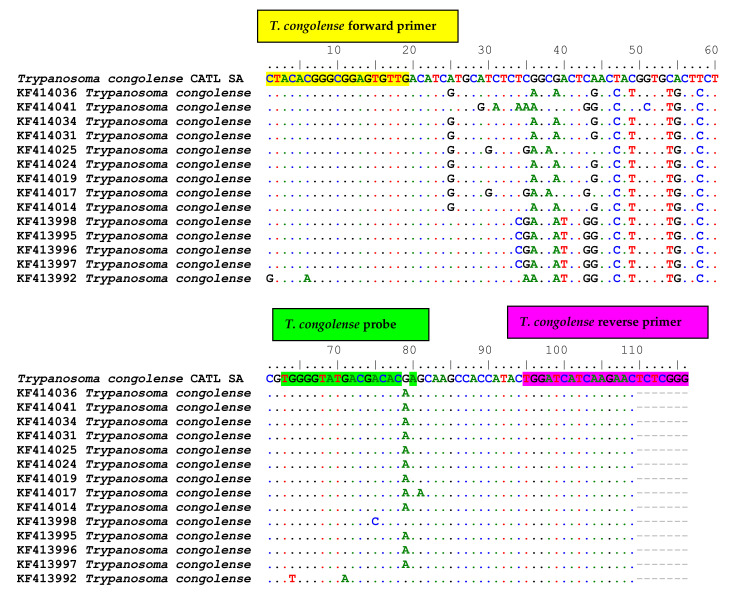
Nucleotide alignment of a section of the *T. congolense* CATL-like gene sequence against published *T. congolense* CATL-like sequences available on GenBank. The sequence of the species-specific forward primer is highlighted in yellow, while the genus-specific reverse primer is highlighted in pink and the species-specific *T. congolense* probe sequence is indicated in green.

An evaluation of 60 cattle samples showed that the animals were infected with *T. congolense*, and *T. theileri* parasites. In addition, the CATL qPCR assays could detect parasite DNA in 24.5% of samples that were BCT negative. Confirming that qPCR is far more sensitive than BCT. The developed CATL qPCR assays detected 3 more *Trypanosoma* infections in the 60 cattle samples than the ITS qPCR. All the ITS PCR and ITS qPCR *T. congolense* positive samples were confirmed by the *T. congolense* CATL qPCR assay. Thirteen (21.7%) of the cattle field samples tested positive for *T. theileri* with Ct values ranging between 30.7 and 38.32 (Ct ≥ 39 regarded as negative). Two of these samples were detected at Ct values above 37.97 (10^2^ copies/µL) indicating very low parasitemia and possibly false positives. In contrast to the ITS qPCR assay which detected *T. brucei* infections, the *T. brucei* CATL qPCR assay did not detect any *T. brucei* infections in the cattle samples. Most of the samples (66.7%) identified as *T. brucei* positive by ITS qPCR assay, were *T. theileri* positive using the CATL qPCR assays, confirming that the *T. brucei* ITS qPCR probe cross-reacts with *T. theileri*. However, 33.3% of the *T. brucei* ITS qPCR positive samples were not detected by any of the CATL qPCR assays. An analysis of the *T. theileri* CATL gene sequence obtained in this study against a more comprehensive list of published sequences available on GenBank indicated 4 nucleotide base-pair differences in the reverse primer sequence which explains the efficiency of the *T. theileri* CATL qPCR assay in detecting the *T. brucei* ITS qPCR positive samples (Appendix A: Figure A2). Redesigning this primer would be required in order to improve the efficiency of the *T. theileri* CATL qPCR assay. This would entail amplifying and confirming the sequences of the full-length CATL gene from *T. brucei* positive samples using the published primers TthCATL1 and DTO155 [19,24,25]. Additionally, nucleotide alignments of the *T. congolense* and *T. brucei* CATL gene sequences obtained in this study, to published *T. congolense* and *T. brucei* CATL gene sequences, respectively, showed high sequence conservation in the regions where the CATL qPCR primers and probes were designed, confirming that if the samples detected by ITS qPCR assay were indeed positive for *T. brucei*, then the *T. brucei* CATL qPCR assay would have detected them (Appendix A: Figure A3 and Figure A4).

A study on AAT prevalence in KZN, reported the occurrence of both *T. brucei* and *T. theileri*, and reported the occurrence of *T. congolense* and *T. theileri* as single infections in cattle [18]. In this study, the developed CATL qPCR assays confirmed the presence of *T. theileri* in NKZN. However, in contrast to the findings reported by Taioe [18], the CATL qPCR assays detected mixed infections with two parasites. Although the current study documented *T. congolense* as the most abundant pathogenic trypanosome, the non-pathogenic *T. theileri* was the overall most abundant parasite. Application of highly sensitive and specific tools, such as the CATL qPCR assays, in AAT epidemiological studies, might reveal much higher *T. theileri* and *T. congolense* prevalence rates in NKZN. It is possible that the prevalence of AAT in South Africa might have been under-reported in previous years, as the diagnostics tools that have been used (such as BCT and conventional PCR) are of less sensitivity than the real-time PCR tool.

Research on AAT occurrence and prevalence in goats in NKZN is limited. Although the study by Taioe [18], reported the detection of trypanosomes in both cattle and tsetse flies, the detection of any *Trypanosoma* infections in goats using a PCR assay targeting the ITS gene was consistently negative. In the present study, the BCT and ITS PCR assays failed to identify trypanosome infections in goats. However, the CATL qPCR assays detected *T. congolense* infections in 9 goat samples, while none of the goats tested positive for *T. theileri* and/or *T. brucei*.

A study conducted by Simukoko [39] to determine the significance of goats, cattle, and pigs in AAT epidemiology on the eastern plateau of eastern Zambia, indicated that parasitological analysis was unable to detect AAT infections in goats. However, 3.3% AAT prevalence was determined in goats using molecular analysis, with *T. congolense* being the major parasite of infection. They concluded that molecular analysis was a better diagnostic tool in detecting trypanosome parasites. Simukoko [39] also observed that when the majority of livestock present in an area is cattle, then the tsetse flies will be more attracted to feed on cattle than other small ruminants. Thus, infections rates are likely to be higher in cattle than in small ruminants such as goats. *Trypanosoma* infections in goats have also been shown to be less virulent than in cattle [40]. The study by Mahmoud [40] showed goats, experimentally infected with *T. congolense*, recovered from chronic infections, yet calves experimentally infected with the same *T. congolense*, died from trypanosomosis infections. However, the presence of trypanosomes should not be overlooked since trypanosomes coupled together with other constraining factors such as poor nutrition, helminth infections, can cause serious chronic infections in goats.

The detection of *Trypanosoma* infections in goats using qPCR assays indicates the superior sensitivity of qPCR over conventional parasitological techniques and conventional PCR assays. The observed high Ct values are indicative of low parasitemia, which explains why these parasites could not be detected using BCT and ITS PCR. Low parasitemia of trypanosomes in goats was observed in a molecular diagnostics study in Zambia [41]. The study used PCR assays that targeted the ITS1 gene and the CATL gene of *T. vivax* isolates and recorded no significant differences in the PCV values of the *Trypanosoma* infected and non-infected goats. It was concluded that AAT in goats does not usually show clinical signs [41].

The probability of more *Trypanosoma* species circulating in goats in NKZN cannot be ruled out as only a few goat samples were screened by the qPCR. Several studies have shown goats can be naturally infected with *T. brucei* [42,43,44], *T. cruzi* [45], *T. vivax* [44,46], *T. evansi* [46,47], *T. theodori,* and *T. uniforme* [48,49]. In NKZN, goats are likely to be naturally infected with *T. congolense*, *T. vivax,* and possibly *T. brucei* as these are the major trypanosomes reported in the area.

## 4. Materials and Methods

### 4.1. Field Sample and Buffy Coat Technique

A total of 39 goats and 60 cattle were sampled from northern KwaZulu-Natal (KZN) between March and April 2019. Blood collection from the cattle and goats was conducted at Mvutshini, Ocilwane, and Ekuphindisweni dip tanks. Blood was collected from the tail veins of adult cattle and the jugular veins of adult goats, into 10 mL vacutainer tubes containing EDTA (BD Vacutainer^®^; BD, Plymouth, UK).

The blood samples were investigated for the presence of *Trypanosoma* parasites using the buffy coat technique (BCT). A small aliquot of the collected blood from each sample was transferred to microhaematocrit centrifuge capillary tubes that were sealed with wax plates (Marienfeld, Lauda-Königshofen, Germany) and centrifuged in a haematocrit centrifuge. Centrifugation was done at 11,000× *g* for 5 min to separate the plasma and blood cells. The packed cell volume (PCV) for all the samples was determined using a haematocrit tube reader, and cattle with PCV values of 24% or less were considered anemic [50].

The prevalence of *Trypanosoma* infection was calculated at each dip tank as the percentage of the animals with trypanosome infections (herd average prevalence) [28]. The average anemia for both cattle and goats at each dip tank was calculated as the average of all anemic animals (PCV ≤ 24%) at the particular dip tank [8]. The herd average PCV (HA-PCV) was calculated for both cattle and goats as the average PCV at each sampling site [28]. The above values are important in the study as they are herd health status indicators [8,51].

After determining the PCV, the buffy coat layer was extruded onto a microscope slide, and slides were examined for motile trypanosomes using a compound microscope at 40-times magnification. *Trypanosoma* species identification was based on parasite motility and morphology.

### 4.2. Molecular Identification of Trypanosomes Using ITS PCR

All samples (Goat: n = 384 and Cattle: n = 60) were tested for the presence of *Trypanosoma* parasite DNA using the conventional polymerase chain reaction (PCR) targeting the ITS region of the rRNA gene. Briefly, published ITS1 CF forward (5′CCGGAAGTTCACCGATATTG3′) and ITS1 BR reverse (5′TGCTGC GTTCTTCAACGAA3′) primers were used to produce amplicons of sizes between 250–710 bp, depending on the trypanosome species [52]. The PCR was carried out in a reaction volume of 25 μL containing 12.5 μL One Taq^®^ Quick-Load^®^ 2× Master Mix with Standard Buffer (New England, Biolabs^®^ Inc., Ipswich, MA, USA), 10 µM of ITS1 CF primer, 10 µM of ITS1 BR primer, 9 μL of nuclease-free water and 2.5 μL extracted DNA. Reactions were performed using GeneAmp^®^ PCR System 9700 (Applied Biosystems, Johannesburg, South Africa). The thermocycling conditions included an initial denaturation of 3 min at 94 °C, followed by 30 cycles of 30 s at 94 °C, 30 s at 55 °C, and 30 s at 72 °C, with a final extension of 10 min at 72 °C. DNA from *T. congolense*, *T. brucei,* and *T. vivax* were used as positive controls and the negative control was a no template control. The amplicons were visualized by 2% agarose gel electrophoresis at 120 V for 30 min.

### 4.3. ITS Real-Time PCR Assay

Cattle DNA samples were screened for the presence of *Trypanosoma* using published genus-specific PCR primers targeting the ITS1 gene [14]. One set of universal primers and three probes, each specific for *T. congolense*, *T. vivax,* and *T. brucei* were designed to amplify a 120 bp region within the ITS1 gene (Table 4). The assay was modified for use on the LightCylcer^®^ 480 PCR platform at the ARC-OVR Epidemiology, Parasites, and Vectors Department. For the simultaneous detection of all three parasites in a multiplex assay, each of the species-specific probes was labeled with different fluorescent dyes (Table 4).

Briefly, quantitative real-time PCR assays (qPCR) were performed using the LightCycler^®^ 480 PCR System (Roche Molecular Diagnostics, Mannheim, Germany). Reactions were performed in a 20 μL volume containing 10 μL KAPA Probe Fast Universal Master Mix (Kapa Biosystems, Cape Town, South Africa), 0.9 μM Tryps_KS-forward primer, 0.9 μM Tryps_KS-reverse primer, 0.25 μM of each of the species-specific probes, 2.5 μL of target DNA and 3.5 μL of nuclease-free water. Cycling conditions were as follows: enzyme activation at 95 °C for 30 s followed by 45 cycles of 5 s at 95 °C and 40 s at 68 °C. Data were analyzed with the LightCycler^®^ 480 software using the Second Derivative Maximum Method.

### 4.4. Design of Primers and TaqMan MGB™ Probes

A multiple sequence alignment of 36 *Trypanosoma* spp. cathepsin L (CATL) reference sequences retrieved from GenBank (https://www.ncbi.nlm.nih.gov/) (accessed on 17 October 2020), was created using the MUSCLE algorithm. A TaqMan minor groove binder (MGB™, Applied Biosystems, Waltham, MA, USA) real-time PCR assay was designed for *T. congolense*, *T. brucei,* and *T. theileri* using the Primer Express software v3.0 (Applied Biosystems, Waltham, MA, USA) and evaluated using BLAST-n under default algorithm parameters. Three species-specific forward primers; one genus-specific reverse primer and three TaqMan MGB™ probes were designed (Table 5) to amplify 116 bp, 98 bp, and 137 bp fragments of the CATL-like gene from *T. congolense*, *T. brucei,* and *T. theileri*, respectively.

Quantitative real-time polymerase chain reaction (qPCR) assays were performed in MicroAmp™ optical 96-well plates using the StepOnePlus™ real-time instrument (Applied Biosystems™, Waltham, MA, USA). Reactions were prepared in a final volume of 20 µL containing 10 µL KAPA Probe Fast Universal Master Mix (Kapa Biosystems, Cape Town, South Africa), 0.9 µM of each CATL species-specific forward primer and genus-specific reverse primer, 0.25 µM TaqMan MGB™ species-specific probe, and 2.5 µL target DNA. The qPCR cycling conditions were as follows: activation at 95 °C for 20 s followed by 40 cycles of 1 s at 95 °C and 30 s at 60 °C. Results were analyzed using the StepOnePlus™ software (v2.3).

### 4.5. Analytical Sensitivity of CATL qPCR Assays

Plasmid standards were generated for each parasite species to determine the sensitivity of each CATL qPCR assay. Species-specific positive control DNA of *T. theileri*, *T. brucei*, and *T. congolense* was obtained from UP DVTD molecular diagnostics laboratory and the CATL-gene was amplified from each control using the species-specific forward and genus-specific reverse primers described previously (Table 5). Reactions were set up in a final reaction volume of 25 μL containing 12.5 μL One Taq^®^ Quick-Load^®^ 2× Master Mix (New England, Biolabs^®^ Inc., Ipswich, MA, USA), 10 µM of CATL species-specific forward primer, 10 µM of CATL genus-specific reverse primer, 9 μL of nuclease-free water and 2.5 μL of the DNA control. The GeneAmp^®^ PCR System 9700 (Applied Biosystems, Johannesburg, South Africa) was used for amplification and the cycling conditions included an initial denaturation at 94 °C for 3 min, followed by 30 cycles of 94 °C for 30 s, 56 °C for 30 s, 72 °C for 30 s, with a final extension step at 72 °C for 10 min. The amplicons were gel purified using the PureLink^®^ Quick Gel Extraction and PCR Purification Combo Kit (Thermo Scientific, Waltham, MA, USA) and cloned into the pJET1.2/blunt Cloning Vector, according to the manufacturer’s instructions. Four recombinant clones per *Trypanosoma* species were selected, plasmid DNA was isolated using the High Pure Plasmid Isolation Kit (Roche, Switzerland), and plasmid inserts were sequenced pJET1.2 forward and reverse primers to confirm the species of each cloned product. Plasmid DNA was sent to Inqaba Biotechnologies (South Africa) for capillary DNA sequencing. The plasmid inserts were sequenced using pJET1.2 forward sequencing primer (5′-CGACTCACTATAGGGAGAGCGGC3′) and pJET1.2 reverse sequencing primer (5′-AAGAACATCGATTTTCCATGGCAG3′). Sequences were assembled and edited using the CLC Genomics Workbench (CLC Bio version 8.0.1). Multiple sequence alignments were performed using the MUSCLE algorithm and sequences were queried against previously published CATL gene sequences using BLAST. Based on the sequences, for each *Trypanosoma* species, one recombinant clone was selected, and the DNA concentration was determined using the Xpose DNA/RNA Analysis (Trinean, Unchained Labs, Gent, Belgium). The copy number for each *Trypanosoma* parasite species plasmid construct was calculated and the concentration of each plasmid construct was adjusted to 10^9^ copies/µL. A ten-fold serial dilution (10^9^–10^0^ copies/µL) was prepared and qPCR amplification of each standard dilution series was repeated in triplicate. Linear regression curves were generated by plotting the mean cycle threshold (Ct) values against the mean log copy number. The formula E = 10^1/−s^ − 1 was used to determine the efficiency of each assay.

### 4.6. Analytical Specificity of CATL qPCR Assays

The intraspecies analytical specificity was determined using DNA samples from other *Trypanosoma* species expected to occur in livestock. Similarly, the interspecies specificity of each assay was determined by testing DNA samples from other parasites known to infect cattle, such as *B. bovis, B. bigemina,* and *T. parva*.

### 4.7. Evaluation of Field Samples Using CATL qPCR Assays

DNA was extracted using DNeasy^®^ Blood and Tissue Kit (Qiagen, Hilden, Germany) following the manufacturer’s instructions. The extracted DNA from field samples collected from cattle (n = 60) and goats (n = 39) and were used to evaluate the diagnostic ability of each of the developed *Trypanosoma* CATL qPCR assays in detecting parasite DNA.

### 4.8. Data Analysis

A Microsoft^®^ Excel spreadsheet was used for data entry and the data was transferred to the Statistical Package for Social Sciences (SPSS) version 26.0 software for statistical analysis. One-way analysis of variance (ANOVA) was used to analyze differences in the HA-PCV of goats and cattle between the three dip tanks. Tukey post hoc test was used to test for the difference in means among dip tanks. The independent t-test was used to test the association between: (a) the average PCV of infected and non-infected animals (b) the average PCV of anemic and non-anemic animals. The Fisher’s exact test was performed to determine differences between BCT, ITS PCR, ITS qPCR, and CATL qPCR. Confidence intervals of 95% and a *p*-value of 0.05 were used. The formula “infection rate” = ((number of cattle infected)/(number of cattle sampled)) × 100 was used to calculate the *Trypanosoma* infection rate.

## 5. Conclusions

Findings from the molecular and parasitological survey of African animal trypanosomosis (AAT) in goats and cattle showed the pathogenic *T. congolense* is prevalent at the three dip tanks sampled in northern KwaZulu-Natal. This finding confirms the results of previous studies that observed *T. congolense* as the major pathogenic trypanosome in the region. Although *T. brucei* was reported in the survey, these samples were considered suspect positive samples due to their low parasitemia and scarcity of the parasite in NKZN. *Trypanosoma vivax* was not detected in this study, which confirms its low occurrence in the region.

Comparison of the three diagnostic tools used in the survey revealed that there was no significant difference in infection rates detected by both the internal transcribed spacer (ITS) polymerase chain reaction (PCR) and by the buffy coat technique (BCT). However, the ITS quantitative real-time PCR (qPCR) assay proved to be a better diagnostic tool in detecting trypanosomes than the ITS PCR and BCT, showing higher sensitivity of real-time PCR assays.

In this preliminary study, the three TaqMan MGB™ qPCR assays were designed to target the cathepsin L-like (CATL) gene of *T. congolense*, *T. theileri,* and *T. brucei*, successfully amplified DNA of their respective target trypanosomes. The assays also reported the first molecular diagnosis of *T. congolense* in goats, in South Africa. This finding demonstrates that goats contribute to maintaining and spreading *Trypanosoma* infections to other livestock. The assays further illustrated the presence of the non-pathogenic *T. theileri* in most of the cattle samples that were previously described as *T. brucei* positive by the ITS qPCR. However, the inability of the *T. theileri* the CATL qPCR assay in detecting samples that tested positive using the *T. brucei* ITS qPCR indicated either a lack of specificity or reduced efficiency of the *T. theileri* CATL qPCR assay. Further investigation of published CATL gene sequences, that included a sequence from our *T. theileri* positive control isolate, confirmed nucleotide differences in the *T. theileri* reverse primer region, explaining the failure of the assay in detecting ITS PCR positive samples. A major limitation of this work was that the qPCR amplicons from either locus (CATL and ITS) were not sequenced to confirm the specificity of the developed CATL qPCR assays. Future work should, therefore, include amplifying, cloning, and sequencing the CATL, ITS1, and 18S rRNA genes from positive samples, with a view to redesigning the reverse primer sequence and re-evaluating the efficiency and specificity of the *T. theileri* CATL qPCR assay. Our preliminary study was further limited by the small number of samples that were investigated and screening a larger sample population may give a more accurate depiction of the feasibility of using these tests for diagnostic screening.

Despite these limitations, the CATL qPCR assays showed better detection of trypanosomes in the cattle and goat field samples, than the ITS qPCR assay, ITS PCR, and BCT. It would also be interesting to evaluate the CATL qPCR assays in duplex or multiplex format. Highly sensitive diagnostics tools, such as real-time PCR assays, should be used in AAT studies for a better understanding of the disease status, and should be used to gather deeper knowledge on *Trypanosoma* diversity in South Africa.

## Figures and Tables

**Table 1:** Trypanosome infection rates, HA-PCV, and HAA in cattle at the three dip tanks.

Site Name	Number of Cattle	Infection Rate (%)	HA-PCV (% ± S.D)	HAA (%)
Ocilwane	20	5	28.9 ± 4.50 ^a,b^	15
Mvutshini	20	15	32.4 ± 3.98 ^b^	10
Ekuphindisweni	20	15	28.9 ± 4.50 ^a,b^	20
Significance		*p* > 0.05	*p* < 0.05	*p* > 0.05

^a^ The significant difference between Ocilwane and Ekuphindisweni HA-PCV, *p* = 1.00. ^b^ The significant difference between Ocilwane/Ekuphindisweni and Mvutshini HA-PCV, *p* = 0.035.

**Table 2:** *Trypanosoma* CATL qPCR assays specificity.

Species Name	*T. Brucei*-Specific qPCR Assay Ct	*T. Congolense*-Specific qPCR Assay Ct	*T. Theileri*-Specific qPCR Assay Ct
*B. bovis*	negative	negative	negative
*B. bigemina*	negative	negative	negative
*T. parva*	negative	negative	negative
*T. vivax*	negative	negative	negative
*T. brucei*	26.21	negative	negative
*T. congolense*	negative	23.74	negative
*T. theileri*	negative	negative	25.71

**Table 3:** Comparison of diagnostic techniques used in detecting trypanosomes.

	**Cattle Samples (n = 60)**				**Goats’ Samples (n = 39)**		
	BCT	ITS PCR	ITS qPCR	CATL qPCR	BCT	ITS PCR	CATL qPCR
*T. congolense*	7	5	6	8	0	0	9
*T. brucei*	0	0	12	0	0	0	0
*T. vivax*	0	0	0	ND	0	0	0
*T. theileri*	0	ND	ND	13	0	ND	0
Total positive	7	5	18	21 *	0	0	9
Negative	53	55	42	40	39	39	30
Significance	*p* = 0.001				*p* = 0.000		

^*^ One sample had mixed infections of *T. congolense* and *T. theileri*. ND: Test was not done.

**Table 4:** Universal primers and genus-specific ITS1 real-time probes for detecting AAT [14].

Name	Primer/Probe	Product Size
Tryps_KS-for	5′-CGT GTC GCG ATG GAT GAC TT-3′	120 bp
Tryps_KS-rev	5′-CAA ACG GCG CAT GGG AG-3′	120 bp
Tryps_KS-T.cong-p	CY5 5′-TTG CAG AAT CAT CAC ATT GCC CAA TCT TTG-3′ BHQ1	
Tryps_KS-T.brucei-p	FAM 5′-TGC GAT AAG TGG TAT CAA TTG CAG AAT CAT TTC A-3′ BHQ1	
Tryps_KS-T.vivax-p	HEX 5′-ATG ACC TGC AGA ACC ACT CGA TTA CCC AGT-3′ BHQ1	

**Table 5:** Primers and probes designed to amplify *Trypanosoma* CATL-like gene.

Name	Primer/Probe	Primer/Probe Length (bp)	Product Size
TcongCATL_fwd	5′-CTA CAC GGG CGG AGT GTT G-3′	19	116 bp
TtheilCATL_fwd	5′-CGA CGC CAA CAG CTT CCT-3′	18	137 bp
TbrucCATL_fwd	5′-GACTTCATGCACCTCCGAGC-3′	20	98 bp
TrypsCATL_reverse	5′-CCC GAG AGT TCT TGA TGA TCC A-3′	22	
TcongCATL_probe	FAM 5′-TGG GGT ATG ACG ACA CAA-3′ MGB	18	
TtheilCATL_probe	VIC 5′-GGC TAC GAC GAC AGC A-3′ MGB	16	
TbrucCATL_probe	NED 5′-GAT AAT AGC AAT CCA CCC-3′ MGB	18	

## Data Availability

Not applicable.
